# Comparing single‐target and multitarget approaches for postoperative circulating tumour DNA detection in stage II–III colorectal cancer patients

**DOI:** 10.1002/1878-0261.13294

**Published:** 2022-08-18

**Authors:** Tenna Vesterman Henriksen, Thomas Reinert, Mads Heilskov Rasmussen, Christina Demuth, Uffe Schou Løve, Anders Husted Madsen, Kåre Andersson Gotschalck, Lene Hjerrild Iversen, Claus Lindbjerg Andersen

**Affiliations:** ^1^ Department of Molecular Medicine Aarhus University Hospital Denmark; ^2^ Department of Clinical Medicine Aarhus University Denmark; ^3^ Department of Surgery Regional Hospital Viborg Denmark; ^4^ Department of Surgery Regional Hospital Herning Denmark; ^5^ Department of Surgery Regional Hospital Horsens Denmark; ^6^ Department of Surgery Aarhus University Hospital Denmark

**Keywords:** circulating tumour DNA, colorectal cancer, liquid biopsy, residual disease

## Abstract

Circulating tumour DNA (ctDNA) detection for postoperative risk stratification in cancer patients has great clinical potential. However, low ctDNA abundances complicates detection. Multitarget (MT) detection strategies have been developed to increase sensitivity. Yet, empirical evidence supporting performance gains of MT vs. single‐target (ST) strategies in a postoperative setting is limited. We compared ctDNA detection in 379 paired plasma samples from 112 stage II–III colorectal cancer patients by ST digital PCR and MT sequencing of 16 patient‐specific variants. The strategies exhibited good concordance (90%, Cohen's Kappa 0.79), with highly correlated ctDNA quantifications (Pearson *r* = 0.985). A difference was observed in ctDNA detection preoperatively (ST 72/92, MT 88/92). However, no difference was observed immediately after surgery in recurrence (ST 11/22, MT 10/22) or nonrecurrence (both 2/34) patients. In serial samples, detection was similar within recurrence (ST 13/16, MT 14/16) and nonrecurrence (ST 3/49, MT 1/49) patients. Both approaches yielded similar lead times to standard‐of‐care radiology (ST 4.0 months, MT 4.1 months). Our findings do not support significant performance gains of the MT strategy over the ST strategy for postoperative ctDNA detection.

## Graphical abstract

Circulating tumour DNA (ctDNA) is potentially useful for postoperative risk stratification, but its detection is challenging. To examine whether tracking multiple targets improves detection, we analysed paired plasma samples from colorectal cancer patients by tracking one or 16 targets. We observed no difference in postoperative ctDNA detection, concluding that widely available single‐target analyses can provide equally good results for recurrence‐risk stratification.

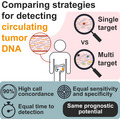

**Abbreviations**
cfDNA: cell‐free DNA
ctDNA: circulating tumour DNA
CRC: colorectal cancer
ddPCR: droplet digital PCR
GE: genome equivalents
MT: multitarget
mPCR: multiplex PCR
NGS: next generation sequencing
NTC: no template control
QC: quality control
ST: single‐target

## 1 Introduction

The surging interest in detecting circulating tumour DNA (ctDNA) seen in recent years is driven mainly by the potential applications for early cancer detection and postoperative minimal residual disease detection [1, 2, 3, 4, 5, 6, 7, 8]. Tumour‐informed ctDNA analyses are currently under investigation in randomized controlled trials for postoperative risk stratification and residual disease monitoring in cancer patients [9]. In these applications, sensitivity is key, as patients with residual disease after curatively intended surgery have a very low tumour burden. Accordingly, the number ctDNA fragments containing a given genomic position is extremely low [10, 11] and vulnerable to sampling stochasticity. To mitigate this challenge, multitarget (MT) strategies have been developed in the expectation that tracking multiple targets in the plasma will increase the likelihood of sampling a sufficient number of informative tumour DNA fragments to call ctDNA when present. However, depending on the technology, MT tracking has potential disadvantages including increased costs, need for additional plasma, additional optimization parameters, and increased analytical complexity. For future clinical application, these disadvantages should be outweighed by a performance increase, yielding a substantial benefit to the patient. As of yet, the empirical evidence supporting a sensitivity difference between single‐target (ST) and MT strategies in the postoperative setting is limited.

In this study, we aim to answer the question: is more always better? We directly compare two tumour‐informed ctDNA detection approaches: ST droplet digital PCR (ddPCR) and MT multiplex‐PCR (mPCR) next generation sequencing (NGS) (Signatera). As such, we apply both assays to aliquots of the same plasma samples from stage II to III colorectal cancer (CRC) patients. We describe the overall concordance in ctDNA detection and quantification, and we compare performance in postoperative risk stratification and serial recurrence monitoring.

## 2 Materials and methods

### 2.1 Patient selection

Patients were selected from two previously published cohorts of CRC patients [2, 3], with MT analyses on plasma samples, and additional plasma available for ST analysis (Fig. S1). Patients undergoing CRC resections with curative intent were recruited prospectively at six Danish hospitals between May 2014 and December 2018. Patients were treated and monitored according to the Danish Colorectal Cancer Groups National Guidelines. Adjuvant treatment decisions were made by the patient and treating physician without knowledge of ctDNA results. The standard‐of‐care follow up included CT‐scans at 12 and 36 months after surgery. The Committees on Biomedical Research Ethics in the Central Region of Denmark approved the study. The study was performed in accordance with the Declaration of Helsinki and all participants provided written informed consent. An overview of experimental details according to the dMIQE guidelines [12] can be found in Table S1.

### 2.2 Sample collection and extraction

For all patients, tumour biopsies were collected from the resected primary tumour, as either fresh frozen or as formalin fixed and paraffin embedded tissue (FFPE). Blood samples were collected in four K2‐EDTA 10 mL tubes (Becton Dickinson, Franklin Lakes, NJ, USA) and plasma isolated within 2 h by double centrifugation. Buffy coat was collected after the first centrifugation. Approximately, 16 mL plasma was obtained per sample and aliquoted into 4 × 4.5 mL cryotubes (Techno Plastic Products AG, Trasadingen, Switzerland). Plasma and buffy coat were frozen immediately and stored at −80 °C until use.

DNA was extracted from fresh frozen tumour tissue samples using the Puregene DNA purification kit (Gentra Systems, Minneapolis, MN, USA) and from FFPE samples using the QiAamp DNA FFPE tissue kit (Qiagen, Hilden, Germany). Tissue and buffy coat DNA was quantified by the Qubit™ dsDNA BR Assay Kit (ThermoFisher, Waltham, MA, USA).

Cell‐free DNA was purified from two plasma aliquots of ~ 4 mL for each of the ST and MT analyses (~ 8 mL for each analysis, with 6 mL being the required minimum), and eluted in a 60 μL volume with no extraction replicates. No extraction blanks were used. For MT analyses, cfDNA was extracted manually using the QIAamp Circulating Nucleic Acids kit (Qiagen) and quantified using the Quant‐iT High Sensitivity dsDNA Assay Kit (Invitrogen, Waltham, MA, USA). For ST analysis, cfDNA was extracted using the QIAsymphony DSP Circulating DNA Kit (Qiagen) on the QiaSymphony robot (Qiagen), and cfDNA was quantified by ddPCR (Bio‐Rad Laboratories, Hercules, CA, USA), using assays targeting two regions on Chr3 and Chr7 shown to rarely have copy‐number alterations in CRC, as described previously [7]. Extracted cfDNA was either used immediately, or stored at −80 °C until use.

### 2.3 Whole exome sequencing

Data from whole exome sequencing was available from previously published results [2, 3]. In brief, Illumina‐adapter based libraries were generated with a median 500 ng (range: 181–500 ng) of genomic DNA from tumour and buffy coat. Whole exome sequencing (target size ~ 40 Mb) was conducted using the NovaSeq platform at 2 × 100 bp paired‐end sequencing. Tumour and buffy coat DNA samples were sequenced to an average coverage of 180× and 50×, respectively. FastQ files were prepared using bcl2fastq2 and quality checked using fastqc. Reads were mapped to the human reference genome hg19 using burrows–wheeler alignment tool (v.0.7.12) and quality checked using picard and multiqc. To identify sample swaps, the SNP genotype concordance between tumour and matched buffy coat DNA samples was examined.

### 2.4 Multitarget analysis: Signatera

Multitarget cfDNA analysis was conducted with the Signatera mPCR NGS approach, described in detail in previous publications [2, 3], where all results used in this article can be found. In brief, WES data was processed through Natera's proprietary bioinformatics pipeline for identification of clonal somatic single nucleotide variants. A prioritized list of variants from the candidate pool of clonal variants was used to design PCR amplicons targeting 16 clonal mutations for every patient. cfDNA was extracted from 8 mL plasma, and libraries were prepared using up to 66 ng of cfDNA and subjected to end‐repairing, A‐tailing and adapter ligation, followed by amplification and purification of the product using Ampure XP beads (Agencourt/Beckman Coulter, Brea, CA, USA). A 16‐plex targeted PCR was conducted on an aliquot of each library. Amplified, barcoded products were pooled and sequenced with an average > 100 000× raw coverage on an Illumina platform. A sample was called ctDNA positive if ≥ 2 variants were detected based on a previously defined confidence threshold [13]. The ctDNA level for each sample was calculated as the mean ctDNA level (mean tumour molecules per mL plasma) for all 16 targets, including targets without mutations detected.

### 2.5 Single‐target analysis: ddPCR

From WES data, mutation clonality was assessed using variant allele frequencies from mutect2 [14] and estimates of cancer cell purity, tumour ploidy, and allele‐specific copy numbers from bubbletree [15]. Clonal mutations in each patient were compared with our in‐house panel of 100 ddPCR assays, designed to target the most common clonal mutations in CRC. If a patient did not have a clonal mutation within the ddPCR assay panel, plasma was not analysed with ddPCR. Choice of ddPCR target was made independently of and blinded to MT‐target selection.

All ddPCR assays consisted of a primer set amplifying the target region, one probe reporting the mutation and another probe reporting the corresponding wild‐type sequence. Primer/probe sequences and PCR protocols are provided in Table S2. Assays were either custom designed or made‐to‐order from ThermoFisher. primer3 [16] was used to check for mispriming, GC‐content and optimal melting temperatures in custom assays, and sequences were checked for cross reactivity using *in silico* PCR [17]. All amplicons were < 150 bp in length.

ddPCR assays were checked for linearity using a 4‐point dilution series of tumour DNA in a uniform concentration of 3030 genome equivalents (GE) of wild‐type DNA per μL (mutant allele frequencies of: 1%, 0.3%; 0.1%; 0.03%). The optimal elongation temperature was assessed on a five‐point temperature gradient, and selected based on linearity, droplet amplitude and sensitivity.

All ddPCR reactions were carried out in a prepared 22 μL reaction volume, with 20 μL being converted to droplets (approx. 0.834 nL each [18]). The cfDNA was extracted from 8 mL plasma and the eluted DNA volume was 60 μL, of which 54 μL were intended for ctDNA analysis and 4 μL were used for cfDNA quantification. To avoid oversaturation of the droplets with wild‐type DNA, the eluate was divided into multiple ddPCR reactions. To save cfDNA for ctDNA positive patients, we initially analysed 18 μL eluate for a subset of cfDNA samples (105/373). Samples that tested positive were not analysed further, while the remaining cfDNA eluate (36 μL) was analysed for the samples testing ctDNA negative. A water sample was run as a no template control (NTC), an assay‐specific tumour DNA sample carrying the targeted mutation was used as positive control, and a pool of buffy coat DNA from healthy donors were used as a negative control. Additionally, a patient‐specific tumour and buffy coat DNA sample was run alongside the plasma as positive and negative control, respectively. Mastermix was prepared with ddPCR Supermix for probes (No dUTP) (Bio‐Rad; Cat:1863024) and a 20× mix of primers and probes in a 1 : 10 ratio. Droplets were generated on the Automated Droplet Generator (Bio‐Rad), and PCR reactions were run on the S1000 Thermal Cycler (Bio‐Rad). After PCR, plates were kept at 12 °C for 5 h or overnight to ensure maximum droplet stability. Droplets were analysed on a QX200™ Droplet Reader (Bio‐Rad). The average number of droplets analysed per well was 16 948 [standard deviation (SD) 1852], with an average of 0.18 DNA copies per droplet (SD 0.23).

### 2.6 ddPCR data processing

The assay‐specific positive control sample was used to set the threshold for positive droplets using the quantasoft software (v1.7.4; Bio‐Rad, Fig. S2). The threshold was set a predetermined assay‐specific number (Table S2) of standard deviations from the mean amplitude of the positive droplets in the positive control. Quality control (QC) parameters for ddPCR analysis were no signal in NTC; signal in positive control; droplet amplitudes within expected intervals determined from linearity data; and minimum of 8000 droplets in the well. All reaction‐level results passing QC were merged to obtain the sample‐level results.

An assay‐specific noise profile was generated for every assay from false‐positive counts of 94 fractionated buffy coat DNA samples at different DNA concentrations from healthy controls. To determine whether a plasma sample was ctDNA positive, four previously described ddPCR‐calling methods (CASTLE [19], Poisson [19, 20], ALPACA [19, 21], and Dynamic LOB [19]) were used to compare the signal observed in plasma samples to the assay‐specific noise‐profile. A consensus call was made, if ≥ 3/4 callers agreed. If 2/4 callers agreed, the ctDNA call from the CASTLE‐algorithm was used, as this algorithm has previously been demonstrated to be the most robust [19]. The ctDNA level was estimated by the CASTLE algorithm as the error‐corrected number of mutated molecules per mL of plasma.

### 2.7 Statistics

Fraction of ctDNA positive calls were compared by a two‐sided Wilcoxon rank‐sum test and paired data was assessed using the Wilcoxon signed‐rank test. A 95% confidence interval for the fraction of ctDNA positive calls was calculated as a Wilson score interval. Nonpaired binary count data was assessed by a Fisher's exact test. Cohen's kappa was used to compare concurrence between the two approaches. A log–log‐linear regression was used to analyse the ctDNA levels estimated by the two approaches and the Pearson's *r* was used to assess the correlation.

Correlation between ctDNA detection and recurrence‐free survival was evaluated by cox regression. Recurrence‐free survival was measured from the date of surgery until radiological recurrence or end of radiological follow‐up. For serial ctDNA measurements, ctDNA was treated as a time‐dependent covariate.

## 3 Results

### 3.1 Patient cohort

**Fig. 1:**
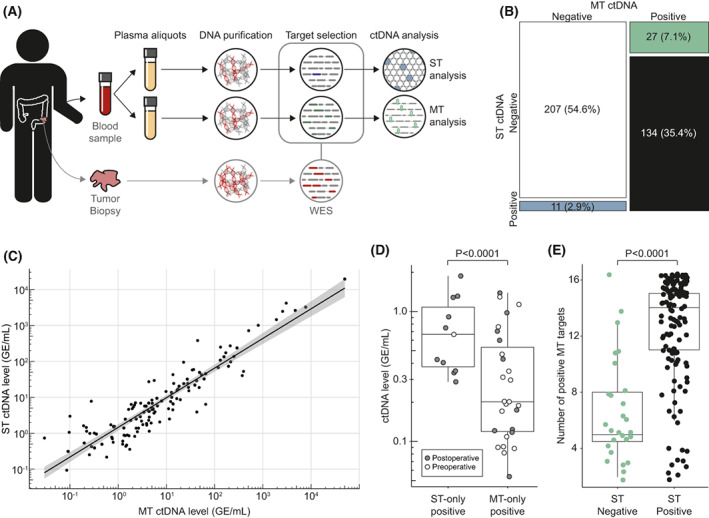
ctDNA call concurrence. (A) Overview of study design. (B) Mosaic plot of samples (*N* = 379) called positive or negative for ctDNA with a ST approach and/or MT approach. (C) Linear correlation between ctDNA level determined by ST and MT in samples called ctDNA positive with both methods (*n* = 134). Shaded area indicates 95% confidence interval for the linear fit. (D) Boxplot of ctDNA level in discordant samples estimated by the approach calling the sample ctDNA positive. Samples were subdivided into ST‐only positive (*n* = 11) and MT‐only positive (*n* = 27), and coloured according to sample timing. (E) Samples detected ctDNA positive by the MT‐approach (*n* = 161). Boxplot of the number of ctDNA‐positive targets detected by the MT approach shown for samples deemed either ctDNA negative (*n* = 27) or ctDNA positive (*n* = 134) with the ST approach. Boxplot whiskers in D and E indicate the 95% confidence interval.

The ST and MT tumour‐informed analyses were carried out on paired plasma aliquots of the same blood samples (Fig. 1A). The two analyses were conducted blinded to each other. A total of 379 blood samples were analysed from 112 patients, of which 27 experienced disease recurrence. The median time to recurrence was 12.2 months [Interquartile range (IQR): 11.6–16.0 months] and the median follow‐up for the 85 nonrecurrence patients was 35.8 months (IQR: 35.4–36.3 months). Patient characteristics are summed up in Table 1, and ctDNA detection data for every plasma sample is given in Table S3.

**Table 1:** Patient demographics and characteristics.

Patients, *n*	112
Age (years), median (range)	69 (43–87)
Sex, *n* (%)	
Female	44 (39.3%)
Male	68 (60.7%)
Stage, *n* (%)	
II	26 (23.2%)
III	86 (76.8%)
Adjuvant chemotherapy, *n* (%)	
No	33 (29.5%)
Yes	79 (70.5%)
Recurrence status, *n* (%)	
No recurrence	86 (76%)
Recurrence	27 (24%)
Tumour site, *n* (%)	
Right colon	54 (48.2%)
Left colon	54 (48.2%)
Rectum	4 (3.6%)

### 3.2 Overall concordance

Overall, a good agreement was observed between the ST and MT results (Fig. 1B), with 90% (341/379) of samples classified the same by both methods (Cohen's Kappa: 0.79, *P* < 0.0001). Both agreed on 134 samples classified as positive. Comparing the estimated ctDNA quantity in these samples, similarly, revealed a good agreement (Fig. 1C, Pearson *r* = 0.985).

### 3.3 Discordant observations

While the majority of samples were classified the same by ST and MT, 11 samples were classified as ctDNA positive exclusively by ST, and 27 were classified as ctDNA positive exclusively by MT (Fig. 1B). The estimated ctDNA level in these discordant samples were in general very low (median 0.3 GE·mL^−1^, IQR 0.2–0.7 GE·mL^−1^). ST‐only positive samples overall had a higher estimated ctDNA level than MT‐only positives (*P* < 0.0001; Fig. 1D). The majority of MT‐only ctDNA positive samples were preoperative samples (17/27, 63%), whereas only 1/11 (9%) ST‐only ctDNA positive samples were collected preoperatively (*P* = 0.0036, Fisher's exact test).

We investigated whether, despite our efforts to ensure otherwise, there were differences in the amount of DNA used for the MT and ST analyses, and particular if this could have influenced ctDNA detection in the samples were ctDNA was detected only by one of the two methods. Overall, there was no difference in the amount of DNA analysed between the two methods in a paired analysis [*n* = 379, ST median 11 738 GE (IQR: 7695–20 710 GE), MT median 14 097 GE (IQR: 9333–20 000 GE), median DNA ratio (ST/MT) 1.0, IQR: 0.70–1.5, *P* = 0.1, Wilcoxon signed‐rank test]. This was similarly the case, in samples testing positive with ST only [*n* = 11, ST median 28 422 GE (IQR: 12619–46 797 GE), MT median 20 000 GE (IQR: 16324–20 000 GE), median DNA ratio (ST/MT) 1.4, IQR: 0.8–2.43, *P* = 0.37, Wilcoxon signed‐rank test]. Moreover, for samples testing positive with MT only [*n* = 27, ST median 10 278 GE (IQR: 6443–16 163 GE), MT median 9418 GE (IQR: 7812–14 095 GE), median DNA ratio (MT/ST) 0.9, IQR: 0.8–1.2, *P* = 0.59, Wilcoxon signed‐rank test].

In samples with ctDNA detected by the MT approach, we compared the number of MT‐positive targets for samples with and without ST‐detected ctDNA. The number of positive targets was higher in the samples with ctDNA detected by ST (median 14; IQR: 11–15) compared with samples without ctDNA detected by ST (median 5, IQR: 5–8; *P* < 0.0001; Fig. 1E).

### 3.4 Preoperative detection

**Fig. 2:**
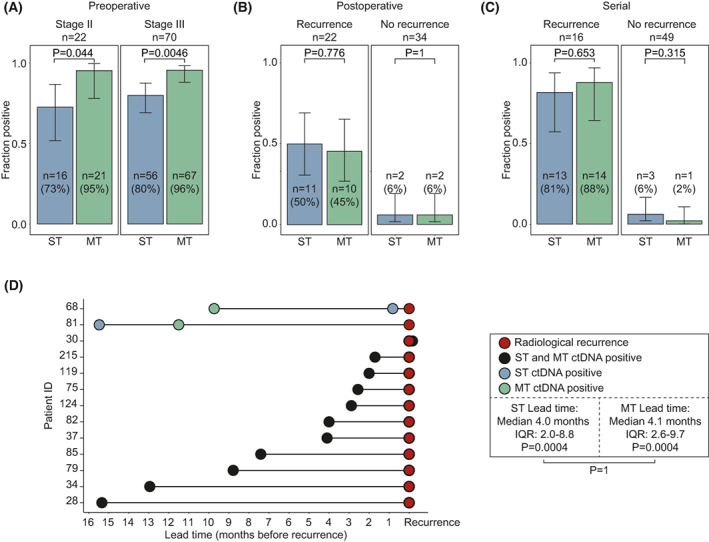
Sensitivity and specificity comparison. (A–C) ctDNA detection for the ST and MT approach respectively. Error bars indicate 95% confidence interval. Fraction positive calls were compared by Wilcoxon rank‐sum test. Results shown stage‐stratified for patients with preoperative samples (*n* = 92, A), recurrence‐stratified for patients with postoperative samples collected within 60 days after surgery before adjuvant treatment (*n* = 56, B), and recurrence‐stratified for patients with serial samples (*n* = 65, C). (D) Lead‐time of ctDNA detection compared with radiological recurrence detection in recurrence patients with ctDNA detected by both the ST and MT approach is serial samples (*n* = 13, see Fig. S3). Lead time from ctDNA detection to radiological recurrence was calculated for both ST and MT approaches and tested using a Wilcoxon signed‐rank test (annotated within the box). Differences between the lead‐time of the two approaches was tested using a Wilcoxon signed‐rank test (annotated outside the box).

We compared the ctDNA detection rates for ST and MT in samples collected preoperatively. Preoperative samples were available for 22/26 stage II patients and 70/86 stage III patients. A higher ctDNA detection rate was observed using MT compared with ST (Fig. 2A) for both stage II (*P* = 0.044) and III patients (*P* = 0.0046).

### 3.5 Detection directly after surgery

Multiple studies have indicated that ctDNA detection immediately after surgery is associated with poor prognosis. Here, we compared ctDNA detection with ST and MT in recurrence and nonrecurrence patients on samples taken within 60 days of surgery and before initiation of adjuvant treatment. Postoperative samples were available for 22/27 patients with recurrence and 34/85 patients without recurrence. There was no difference in the ctDNA detection rate of ST and MT in either recurrence or nonrecurrence patients (Fig. 2B). All nonrecurrence patients with ctDNA detected after surgery received subsequent adjuvant treatment, which may have eliminated residual disease, as we have previously demonstrated [2, 3]. Both detection strategies showed a strong association between ctDNA status and recurrence free survival (Table 2).

**Table 2:** Correlation between ST‐ and MT‐detected ctDNA and recurrence‐free survival.

	HR (95% CI)	*P*‐value^a^
Postoperative ctDNA (*n* = 93)		
ST negative	–	–
ST positive	7.5 (3.1–17.7)	**< 0.0001**
MT negative	–	–
MT positive	6.8 (2.9–16.0)	**< 0.0001**
Serial^b^ ctDNA (*n* = 75)		
ST negative	–	–
ST positive	33.9 (9.3–124.2)	**< 0.0001**
MT negative	–	–
MT positive	67.7 (14.2–322.2)	**< 0.0001**

^a^ Statistically significant *P*‐values (*P* < 0.05) are marked in bold.

^b^ Samples collected serially after end of definitive treatment: treated as time‐varying independent variables.

### 3.6 Serial ctDNA analysis

A subset of the patients was monitored with serial plasma samples after end of definitive therapy (Figs S1 and S3). Cox‐regression analyses, with ctDNA as a time‐dependent covariate, showed that for both ST and MT serial‐ctDNA analysis, ctDNA detection was strongly correlated with recurrence‐free survival (Table 2). There was no statistical difference between the ST and MT ctDNA detection rates for recurrence (*n* = 16) or nonrecurrence patients (*n* = 49) (Fig. 2C).

In nonrecurrence patients, 124 serial plasma samples were analysed in total. Of these, three tested ctDNA positive with the ST strategy and one with the MT strategy. On a sample level, this corresponds to a 97.6% specificity for ST and 99.2% specificity for MT (nonsignificant difference, *P* = 0.316, Wilcoxon rank sum test). The four false positive calls were sporadic, affecting only a single of the serial samples available for the four affected patients (Fig. S3). If patients were called positive without taking the subsequent samples into account, the specificity at the patient level would be 94% (46/49) and 98% (48/49) for ST and MT, which is statistically indistinguishable (Fig. 2C, *P* = 0.315, Wilcoxon rank sum test). In contrast to nonrecurrence patients, multiple consecutive plasma samples tested ctDNA positive in recurrence patients (Fig. S3).

### 3.7 Time to recurrence detection

Finally, we compared lead‐time between ctDNA detected by either ST or MT and radiological detection of recurrence in recurrence patients with serial ctDNA measurements. For the closest comparison, lead‐time was calculated on recurrence patients with ctDNA detected by both methods (*n* = 13; Fig. 2D). The median lead‐times for ctDNA detection compared with CT scans were 4.0 months (IQR 2.0–8.8, *P* = 0.0004) for ST and 4.1 months (IQR: 2.6–9.7, *P* = 0.0004) for MT. In a paired analysis, no difference in time for first ctDNA detection was observed between ST and MT (*P* = 1).

## 4 Discussion

An MT approach to ctDNA detection is expected to be more sensitive than an ST approach. Nevertheless, the simplicity in developing, running and interpreting ST analyses have led to widespread use of these detection strategies. Additionally, multiple clinical trials investigating the utility of ctDNA for allocation of treatment and surveillance resources are currently run using ST strategies, demonstrating the confidence in these analyses [9, 22, 23, 24, 25, 26]. Further, ST‐detection strategies based on ddPCR are often less costly and have shorter turn‐around times compared with most MT strategies [27], providing a good foundation for clinical implementation. Therefore, it is relevant to assess potential performance gains in applying complex MT strategies for ctDNA detection. We have compared the performance between a ST ddPCR approach and a 16‐target mPCR NGS approach. Uniquely, we were able to compare ST and MT ctDNA detection in aliquots of the same blood samples in a large clinical dataset. This allowed us to match ctDNA detection directly in a head‐to‐head comparison, rather than relating detection on a cohort level. Overall, we observed a strong concurrence between the ST and MT results. Estimates of ctDNA level was highly correlated between the two methods, which is in accordance with results from previous studies comparing ddPCR and NGS ctDNA quantification in metastatic patients [11, 28].

Tumour‐informed ctDNA detection approaches have great potential as tools for risk stratification after surgery and serially during follow‐up. As postoperative ctDNA is often found in extremely low abundance, sensitivity for low ctDNA levels is a crucial performance parameter. Previously published data show that ctDNA levels increase from first ctDNA detection until recurrence [2, 3, 8]. If one method detects ctDNA later than the other does, this indicates a difference in sensitivity for low ctDNA levels. In our comparison, no difference was observed in the time to first ctDNA detection between the present ST and MT approaches. Further, our results showed only minor differences, if any at all, in sensitivity and specificity of the two approaches, both immediately after surgery and serially during follow‐up. This indicates equal applicability for early recurrence detection and postoperative recurrence risk assessment.

Postoperative ctDNA detection immediately after surgery has been proposed as a means to guide adjuvant treatment decisions. Currently, multiple clinical de‐escalation trials are investigating whether adjuvant treatment can be removed from ctDNA negative patients [24, 25, 29, 30]. This setting requires a high sensitivity, as a false‐negative call may result in a patient not being offered the necessary treatment. The two ctDNA detection approaches evaluated here showed similar sensitivities immediately after surgery (45–50%). These sensitivities may be too low for de‐escalation studies, though they are comparable to or higher than sensitivities reported elsewhere [2, 3, 5, 31]. The clinical utility in using ctDNA for guiding adjuvant treatment decisions thus needs to be demonstrated in larger, prospective studies.

During serial monitoring the two approaches showed statistically indistinguishable specificities (> 97.6%). The specificities we observed are comparable to that of previously reported ctDNA detection strategies using ST or MT methods [2, 3, 6, 32, 33]. The few false‐positive calls were always sporadic singular events, which contrasted the ctDNA detections in recurrence patients which persisted overtime. Today, the follow‐up after curatively intended resection of stage II–III CRC generally follows a one‐size fits all setup, with CT imaging at fixed intervals offered to all patients. This strategy has been used—despite it being well known that 60–80% of patients will not experience recurrence—because no marker has been able to classify this subset of patients reliably. In line with previous studies, our results support that it may now be feasible and beneficial to introduce ctDNA‐guided risk‐stratified follow up to determine when and to whom to offer CT imaging. To this, we uniquely add evidence that both ST and MT ctDNA detection strategies are well‐suited approaches for the needed serial ctDNA analysis. Compared with the present standard where CT‐imaging is offered to all, the low false positive rate of serial ctDNA analysis will most likely be well tolerated and have minor impact in terms of adverse effects and overuse of resources. In particular, if ctDNA positive patients with no findings on subsequent imaging are reverted back to follow‐up with serial ctDNA analysis. Whether ctDNA guided follow‐up will have the expected clinical benefits and cost‐effectiveness needs to be assessed in randomized trials, such as the ongoing IMPROVE‐IT2 trial [23, 34].

To our knowledge, this is the most comprehensive direct comparison between fully optimized ST and MT strategies for ctDNA detection. A recent publication by Loupakis et al. compared the performance of an ST ddPCR approach with the same MT approach used for our analyses (Signatera) in a subanalysis including metastatic CRC patients with a KRAS mutation (*n* = 21) [35]. The study showed greater sensitivity of the MT approach in postoperatively identifying patients who experienced disease progression compared with the ST strategy [86% (18/21) vs. 33% (7/21)]. These results are in contrast to our postoperative sensitivity estimates, showing equal detection between the two techniques. As the MT strategies were the same, the difference to our results likely reflects the rigor with which our ST‐ddPCR analyses were conducted. In contrast to the Loupakis study, and several other studies using ddPCR for ctDNA detection [28, 36, 37, 38], our targets were carefully selected from WES data after a thorough clonality assessment. While the addition of WES prior to ST analysis increases costs, including it minimizes the risk of tracking a subclonal and noninformative variant. Additionally, we used a statistical consensus caller to model the difference in observed ddPCR signal from the expected assay‐specific noise profile, yielding a highly robust ctDNA call. The necessity of accurate error modelling for robust ddPCR calls have been extensively demonstrated previously [19, 20, 21]. Moreover, it is difficult to evaluate, whether the MT and ST analyses in the Loupakis study were comparable in the amount of sample analysed (i.e., whether the same amount of DNA or plasma was used for the two approaches). A discrepancy in the input amounts may explain the observed call discrepancies. Our results therefore highlight that ST‐detection strategies can perform as well as MT‐detection strategies, if optimized to the same degree and performed on equal amounts of input material.

Our comparison demonstrated a good concordance in sample calls, with only a 10% discordance rate. In some cases, only the ST strategy detected ctDNA. Though not statistically significant, we observed a trend towards higher DNA input in the samples analysed with ST compared with the paired sample testing negative with the MT strategy. Likely, this reflects that the MT analyses are carried out on a maximum of 66 ng DNA (~ 20 000 GE) per standard protocol. Thus, in samples with high cfDNA levels, not all the DNA in the sample will be converted to a result. This was not a problem in samples only detected positive by the MT strategy, where the cfDNA input in general was lower (median 9418 GE in MT‐only positive samples compared with the 20 000 GE as median input for the ST‐only positive samples). The majority of discordance was observed in the preoperative samples, and ctDNA detection here was more prevalent using the MT than the ST approach. Preoperative ctDNA detection with tumour‐informed strategies will seldom be clinically relevant, but is often used as an indication of ctDNA sensitivity. However, as illustrated here, a high preOP detection rate does not necessarily translate into a higher postoperative detection rate nor to shorter “time to ctDNA detection”. In agreement herewith, several previous ctDNA studies have reported lower preoperative detection rates, than observed for the MT strategy used here, but nevertheless reported very similar “time to ctDNA‐detections” [8, 39, 40].

While our observations indicated a significantly lower overall ctDNA level in samples detected only by the MT approach, this does not necessarily reflect a better sensitivity. On the contrary, it may reflect a difference in the way the ctDNA level is calculated. With the ST approach, the ctDNA level is calculated based on a single target. This strategy makes the ST quantification notably vulnerable to sampling stochasticity. Here, the minimal ctDNA level observable is in principle one ctDNA fragment out of all input cfDNA fragments. In the rare situations where the real ctDNA level is below this number, but we by chance sampled a tumour DNA fragment in the collected blood volume, the ST approach will overestimate the ctDNA concentration. The MT approach by contrast calculates the ctDNA level as the mean ctDNA level for all 16 targets, including the targets for which no support was observed in the sample. This corrects the estimated concentrations for sampling stochasticity, which will it inevitably result in the MT ctDNA concentration estimates being lower than the ST‐estimated concentrations in samples with very low ctDNA levels. Either way, samples with low ctDNA levels are most vulnerable to subsampling issues, and the MT approach seems to cope better in this setting. This may well be what is reflected in the higher detection rate observed for the MT approach in preoperative samples.

Though both ctDNA detection strategies described in this article are patient‐specific and tumour‐informed, there are methodological differences beyond the number of targets tracked. A comparison of ST ddPCR and MT ddPCR would have decreased the number of variables in the comparison, enabling a better estimate of the contribution of having multiple markers. However, the patient‐specific panel designed for the MT Signatera strategy is nonetheless similar to what would be done using a digital PCR approach, and the comparison is therefore more pertaining to the number of targets than the underlying laboratory protocol (ddPCR vs. NGS). The MT assay tracks 16 somatic tumour variants, and is thus not representative of sequencing strategies covering multiple hundreds of potential targets. The comparison between ST and MT in this article is thus a comparison of a tumour‐informed preselected ST vs. 16‐target approach, and not a representative comparison of ddPCR and NGS as a whole. Whether other MT strategies for ctDNA detection would outperform our current ddPCR setup cannot be concluded from our comparison. It is possible, that MT strategies involving whole‐genome sequencing [41] or investigating many hundreds of potential targets [42] are more sensitive. However, previously published results using the Signatera MT strategy have demonstrated high sensitivities and specificities in a clinical context, not only for CRC [2, 3] but also breast cancer [43], lung cancer [44], and bladder cancer [4].

While our selected ST assays were patient‐specific, we here—for simplicity—choose only to include patients with a somatic mutation overlapping with our already existing in‐house panel of ddPCR assays targeting the most common mutations in CRC. In a prospective setting, an assay could easily be designed for each patient. In settings with fewer recurrent mutations than CRC, the ability to reuse already existing ddPCR‐assays (an in‐house panel) may be less pronounced. In such settings, the ST and MT approaches would be very similar, as both require tumour profiling and patient‐specific assay design and optimization. Even with tumour profiling and individualized assay optimization the ddPCR strategy is reasonably low cost. The cost‐effectiveness of this strategy is expected to increase with the number of samples analysed for each patient (i.e., serial plasma samples for recurrence monitoring), as the up‐front design and optimization costs are fixed.

While we had the advantage of paired samples, this study has some limitations. Firstly, by ensuring the same amount of material for both analyses, we may have introduced some bias in the cohort. Therefore, the results are most useful for comparative analyses and not as an absolute indication of performance of either method in a prospective cohort. Secondly, while the ST results were established blinded to the MT results, they were generated after the MT results. Ideally, the results should have been generated in parallel and completely independent. Thirdly, the modest number of ctDNA positive samples from recurrence patients, both postoperative and serially, warrants larger studies to confirm our findings.

## 5 Conclusions

Our findings do not support a significant performance gain in choosing a multimarker NGS strategy (Signatera) over a simple single‐marker strategy for postoperative ctDNA detection. Moreover, this study highlights the need to compare approaches in their intended setting; i.e. evaluating a recurrence‐risk marker in postoperative samples. While the ctDNA‐field increases focus on MT strategies, widely available ST analyses do provide good results for recurrence‐risk stratification and can perform on par with at least one current MT strategy for early recurrence detection.

## Conflict of interest

The authors declare no conflict of interest.

## Author contributions

Material was gathered by USL, AHM, KAG, LHI and CLA. TVH conducted the data analyses with help from TR, MHR, CD and CLA. Interpretation was primarily conducted by TVH, TR, MHR, CD and CLA, with input from USL, AHM, KAG and LHI. Manuscript was drafted by TVH and CLA with input and revisions from all coauthors. All authors approved of the submitted manuscript.

### Peer Review

The peer review history for this article is available at https://publons.com/publon/10.1002/1878‐0261.13294.

## Supporting information


**Fig. S1.** Inclusion in sub analyses.

**Fig. S2.** Example of positive and negative test result using single‐target ddPCR against the KRAS_c.35G>T_p.G12V mutation.

**Fig. S3.** Overview of samples included in serial analysis and lead‐time analysis.

**Table S1.** dMIQE2020 checklist.

**Table S2.** Information on all ddPCR assays.

**Table S3.** Raw ctDNA detection data using the ST and MT approaches.

## Data Availability

The data that support the findings of this study are available in the supplementary material of this article.

## References

[1] Klein EA , Richards D , Cohn A , Tummala M , Lapham R , Cosgrove D , et al. Clinical validation of a targeted methylation‐based multi‐cancer early detection test using an independent validation set. Ann Oncol. 2021;32:1167–77. 34176681 10.1016/j.annonc.2021.05.806

[2] Henriksen TV , Tarazona N , Frydendahl A , Reinert T , Gimeno‐Valiente F , Carbonell‐Asins JA , et al. Circulating tumor DNA in stage III colorectal cancer, beyond minimal residual disease detection, towards assessment of adjuvant therapy efficacy and clinical behavior of recurrences. Clin Cancer Res. 2021;28(3):507–17. 34625408 10.1158/1078-0432.CCR-21-2404 PMC9401484

[3] Reinert T , Henriksen TV , Christensen E , Sharma S , Salari R , Sethi H , et al. Analysis of plasma cell‐free DNA by ultradeep sequencing in patients with stages I to III colorectal cancer. JAMA Oncol. 2019;5:1124–31. 31070691 10.1001/jamaoncol.2019.0528 PMC6512280

[4] Christensen E , Birkenkamp‐Demtröder K , Sethi H , Shchegrova S , Salari R , Nordentoft I , et al. Early detection of metastatic relapse and monitoring of therapeutic efficacy by ultra‐deep sequencing of plasma cell‐free DNA in patients with urothelial bladder carcinoma. J Clin Oncol. 2019;37:1547–57. 31059311 10.1200/JCO.18.02052

[5] Tie J , Cohen JD , Wang Y , Christie M , Simons K , Lee M , et al. Circulating tumor DNA analyses as markers of recurrence risk and benefit of adjuvant therapy for stage III colon cancer. JAMA Oncol. 2019;5(12):1710–7. 31621801 10.1001/jamaoncol.2019.3616 PMC6802034

[6] Tie J , Wang Y , Tomasetti C , Li L , Springer S , Kinde I , et al. Circulating tumor DNA analysis detects minimal residual disease and predicts recurrence in patients with stage II colon cancer. Sci Transl Med. 2016;8:1–10. 10.1126/scitranslmed.aaf6219 PMC5346159 27384348

[7] Reinert T , Schøler LV , Thomsen R , Tobiasen H , Vang S , Nordentoft I , et al. Analysis of circulating tumour DNA to monitor disease burden following colorectal cancer surgery. Gut. 2016;65:625–34. 25654990 10.1136/gutjnl-2014-308859

[8] Schøler LV , Reinert T , Ørntoft M‐BW , Kassentoft CG , Árnadóttir SS , Vang S , et al. Clinical implications of monitoring circulating tumor DNA in patients with colorectal cancer. Clin Cancer Res. 2017;23(18):5437–45. 28600478 10.1158/1078-0432.CCR-17-0510

[9] Naidoo M , Gibbs P , Tie J . ctDNA and adjuvant therapy for colorectal cancer: time to re‐invent our treatment paradigm. Cancers (Basel). 2021;13:346. 33477814 10.3390/cancers13020346 PMC7832902

[10] Elazezy M , Joosse SA . Techniques of using circulating tumor DNA as a liquid biopsy component in cancer management. Comput Struct Biotechnol J. 2018;16:370–8. 30364656 10.1016/j.csbj.2018.10.002 PMC6197739

[11] Ding PN , Becker T , Bray V , Chua W , Ma Y , Xu B , et al. Plasma next generation sequencing and droplet digital PCR‐based detection of epidermal growth factor receptor (EGFR) mutations in patients with advanced lung cancer treated with subsequent‐line osimertinib. Thorac Cancer. 2019;10:1879–84. 31414729 10.1111/1759-7714.13154 PMC6775001

[12] dMIQE Group , Huggett JF . The digital MIQE guidelines update: minimum information for publication of quantitative digital PCR experiments for 2020. Clin Chem. 2020;66:1012–29. 32746458 10.1093/clinchem/hvaa125

[13] Coombes RC , Page K , Salari R , Hastings RK , Armstrong A , Ahmed S , et al. Personalized detection of circulating tumor DNA antedates breast cancer metastatic recurrence. Clin Cancer Res. 2019;25:4255–63. 30992300 10.1158/1078-0432.CCR-18-3663

[14] Mutect2. n.d. [cited 2021 Jun 25]. Available from: https://gatk.broadinstitute.org/hc/en‐us/articles/360051306691‐Mutect2

[15] Zhu W , Kuziora M , Creasy T , Lai Z , Morehouse C , Guo X , et al. BubbleTree: an intuitive visualization to elucidate tumoral aneuploidy and clonality using next generation sequencing data. Nucleic Acids Res. 2016;44:e38. 26578606 10.1093/nar/gkv1102 PMC4770205

[16] Primer3 Input. n.d. [cited 2021 Jul 1]. Available from: https://primer3.ut.ee/

[17] UCSC In‐Silico PCR. n.d. [cited 2021 Jul 1]. Available from: https://genome.ucsc.edu/cgi‐bin/hgPcr?wp_target=&db=hg19&org=Human&wp_f=TCTTGTCCTGCTTGCTTACC&wp_r=GAGAATCTCCGCAAGAAAGG&wp_size=4000&wp_perfect=15&wp_good=15&wp_showPage=true&hgsid=604693523_KG6phDfurR9aoQScBQPqhpQ3OzjO

[18] Corbisier P , Pinheiro L , Mazoua S , Kortekaas A‐M , Chung PYJ , Gerganova T , et al. DNA copy number concentration measured by digital and droplet digital quantitative PCR using certified reference materials. Anal Bioanal Chem. 2015;407:1831–40. 25600685 10.1007/s00216-015-8458-z PMC4336415

[19] Henriksen TV , Drue SO , Frydendahl A , Demuth C , Rasmussen MH , Reinert T , et al. Error characterization and statistical modeling improves circulating tumor DNA detection by droplet digital PCR. Clin Chem. 2022;68(5):657–67. 35030248 10.1093/clinchem/hvab274

[20] Milbury CA , Zhong Q , Lin J , Williams M , Olson J , Link DR , et al. Determining lower limits of detection of digital PCR assays for cancer‐related gene mutations. Biomol Detect Quantif. 2014;1:8–22. 27920993 10.1016/j.bdq.2014.08.001 PMC5129438

[21] Vessies DCL , Linders TC , Lanfermeijer M , Ramkisoensing KL , van der Noort V , Schouten RD , et al. An automated correction algorithm (ALPACA) for ddPCR data using adaptive limit of blank and correction of false positive events improves specificity of mutation detection. Clin Chem. 2021;67(7):959–67. 33842952 10.1093/clinchem/hvab040

[22] IMPROVE intervention trial implementing non‐invasive circulating tumor DNA analysis to optimize the operative and postoperative treatment for patients with colorectal cancer – ClinicalTrials.gov. n.d. [cited 2022 Feb 1]. Available from: https://clinicaltrials.gov/ct2/show/NCT03748680 10.1080/0284186X.2019.1711170 31920137

[23] Nors J , Henriksen TV , Gotschalck KA , Juul T , Søgaard J , Iversen LH , et al. IMPROVE‐IT2: implementing noninvasive circulating tumor DNA analysis to optimize the operative and postoperative treatment for patients with colorectal cancer–intervention trial 2. Study protocol. Acta Oncol. 2020;59(3):336–41. 31920137 10.1080/0284186X.2019.1711170

[24] ANZCTR – Registration: DYNAMIC trial. n.d. [cited 2022 Feb 1]. Available from: https://anzctr.org.au/Trial/Registration/TrialReview.aspx?ACTRN=12615000381583

[25] ANZCTR – Registration – DYNAMIC‐III trial. n.d. [cited 2022 Feb 1]. Available from: https://anzctr.org.au/Trial/Registration/TrialReview.aspx?ACTRN=12617001566325

[26] ANZCTR – Registration – DYNAMIC‐RECTAL trial. n.d. [cited 2022 Feb 1]. Available from: https://www.anzctr.org.au/Trial/Registration/TrialReview.aspx?ACTRN=12617001560381

[27] Chakrabarti S , Xie H , Urrutia R , Mahipal A . The promise of circulating tumor DNA (ctDNA) in the management of early‐stage colon cancer: a critical review. Cancers (Basel). 2020;12:2808. 10.3390/cancers12102808 PMC7601010 33003583

[28] Demuth C , Spindler K‐LG , Johansen JS , Pallisgaard N , Nielsen D , Hogdall E , et al. Measuring KRAS mutations in circulating tumor DNA by droplet digital PCR and next‐generation sequencing. Transl Oncol. 2018;11:1220–4. 30086420 10.1016/j.tranon.2018.07.013 PMC6085225

[29] Anandappa G , Starling N , Peckitt C , Bryant A , Begum R , Carter P , et al. TRACC: tracking mutations in cell‐free DNA to predict relapse in early colorectal cancer—a randomized study of circulating tumour DNA (ctDNA) guided adjuvant chemotherapy versus standard of care chemotherapy after curative surgery in patients with high risk stage II or stage III colorectal cancer (CRC). J Clin Orthod. 2020;38:TPS4120.

[30] Taniguchi H , Nakamura Y , Kotani D , Yukami H , Mishima S , Sawada K , et al. CIRCULATE‐Japan: circulating tumor DNA‐guided adaptive platform trials to refine adjuvant therapy for colorectal cancer. Cancer Sci. 2021;112:2915–20. 33931919 10.1111/cas.14926 PMC8253296

[31] Taieb J , Taly V , Henriques J , Bourreau C , Mineur L , Bennouna J , et al. Prognostic value and relation with adjuvant treatment duration of ctDNA in stage III colon cancer: a post hoc analysis of the PRODIGE‐GERCOR IDEA‐France trial. Clin Cancer Res. 2021;27:5638–46. 34083233 10.1158/1078-0432.CCR-21-0271

[32] Parikh AR , Van Seventer EE , Siravegna G , Hartwig AV , Jaimovich A , He Y , et al. Minimal residual disease detection using a plasma‐only circulating tumor DNA assay in patients with colorectal cancer. Clin Cancer Res. 2021;27(20):5586–94. 33926918 10.1158/1078-0432.CCR-21-0410 PMC8530842

[33] Benhaim L , Bouché O , Normand C , Didelot A , Mulot C , Le Corre D , et al. Circulating tumor DNA is a prognostic marker of tumor recurrence in stage II and III colorectal cancer: multicentric, prospective cohort study (ALGECOLS). Eur J Cancer. 2021;159:24–33. 34731746 10.1016/j.ejca.2021.09.004

[34] Circulating tumor DNA analysis to optimize the operative and postoperative treatment for patients with colorectal cancer – intervention trial 2 – full text view – ClinicalTrials.gov. n.d. [cited 2022 Feb 1]. Available from: https://clinicaltrials.gov/ct2/show/NCT04084249 10.1080/0284186X.2019.1711170 31920137

[35] Loupakis F , Sharma S , Derouazi M , Murgioni S , Biason P , Rizzato MD , et al. Detection of molecular residual disease using personalized circulating tumor DNA assay in patients with colorectal cancer undergoing resection of metastases. JCO Precis Oncol. 2021;5:PO.21.00101. 34327297 10.1200/PO.21.00101 PMC8315303

[36] Zhang H , Liu R , Yan C , Liu L , Tong Z , Jiang W , et al. Advantage of next‐generation sequencing in dynamic monitoring of circulating tumor DNA over droplet digital PCR in cetuximab treated colorectal cancer patients. Transl Oncol. 2019;12:426–31. 30562681 10.1016/j.tranon.2018.11.015 PMC6297189

[37] Holm M , Andersson E , Osterlund E , Ovissi A , Soveri L‐M , Anttonen A‐K , et al. Detection of KRAS mutations in liquid biopsies from metastatic colorectal cancer patients using droplet digital PCR, Idylla, and next generation sequencing. PLoS One. 2020;15:e0239819. 33237900 10.1371/journal.pone.0239819 PMC7688175

[38] Bolhuis K , van ‘t Erve I , Mijnals C , Delis‐Van Diemen PM , Huiskens J , Komurcu A , et al. Postoperative circulating tumour DNA is associated with pathologic response and recurrence‐free survival after resection of colorectal cancer liver metastases. EBioMedicine. 2021;70:103498. 34333237 10.1016/j.ebiom.2021.103498 PMC8340125

[39] Tarazona N , Gimeno‐Valiente F , Gambardella V , Zuñiga S , Rentero‐Garrido P , Huerta M , et al. Targeted next‐generation sequencing of circulating‐tumor DNA for tracking minimal residual disease in localized colon cancer. Ann Oncol. 2019;30:1804–12. 31562764 10.1093/annonc/mdz390

[40] Chen G , Peng J , Xiao Q , Wu H‐X , Wu X , Wang F , et al. Postoperative circulating tumor DNA as markers of recurrence risk in stages II to III colorectal cancer. J Hematol Oncol. 2021;14:80. 34001194 10.1186/s13045-021-01089-z PMC8130394

[41] Zviran A , Schulman RC , Shah M , Hill STK , Deochand S , Khamnei CC , et al. Genome‐wide cell‐free DNA mutational integration enables ultra‐sensitive cancer monitoring. Nat Med. 2020;26:1114–24. 32483360 10.1038/s41591-020-0915-3 PMC8108131

[42] McDonald BR , Contente‐Cuomo T , Sammut S‐J , Odenheimer‐Bergman A , Ernst B , Perdigones N , et al. Personalized circulating tumor DNA analysis to detect residual disease after neoadjuvant therapy in breast cancer. Sci Transl Med. 2019;11:eaax7392. 31391323 10.1126/scitranslmed.aax7392 PMC7236617

[43] Magbanua MJM , Swigart LB , Wu H‐T , Hirst GL , Yau C , Wolf DM , et al. Circulating tumor DNA in neoadjuvant‐treated breast cancer reflects response and survival. Ann Oncol. 2021;32:229–39. 33232761 10.1016/j.annonc.2020.11.007 PMC9348585

[44] Abbosh C , Birkbak NJ , Wilson GA , Jamal‐Hanjani M , Constantin T , Salari R , et al. Phylogenetic ctDNA analysis depicts early‐stage lung cancer evolution. Nature. 2017;545:446–51. 28445469 10.1038/nature22364 PMC5812436

