# Supplementary material for: Comparing single‐target and multitarget approaches for postoperative circulating tumour DNA detection in stage II–III colorectal cancer patients
Source: Mol Oncol. 2022 Aug 18;16(20):3654–65. doi: 10.1002/1878-0261.13294 (PMC9580876; doi:10.1002/1878-0261.13294)
Supplement: Supplementary file 1 — Fig. S1. Inclusion in sub analyses. [file MOL2-16-3654-s003.pdf]

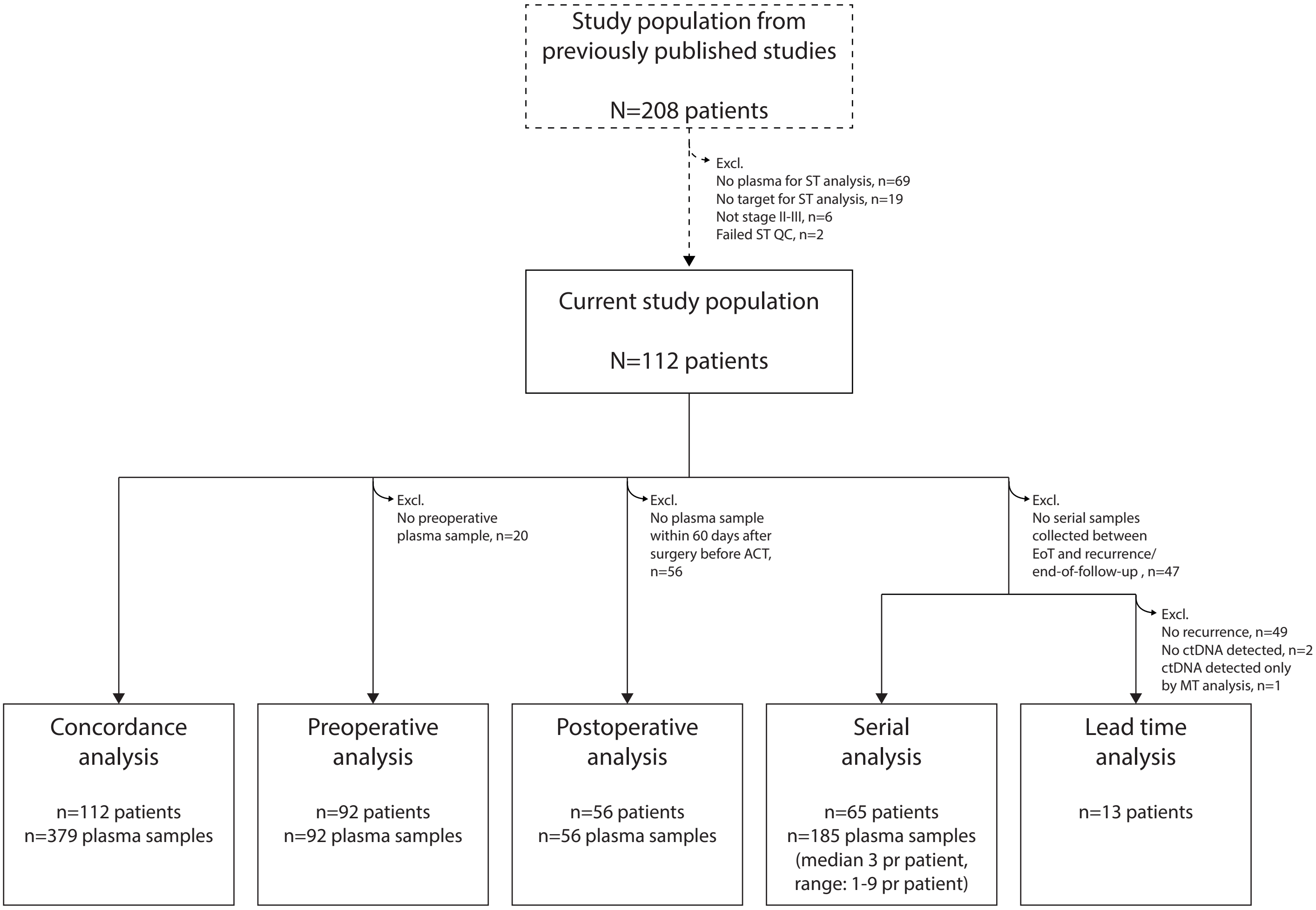

Supplementary Figure S1 – Inclusion in sub analyses. Overview of number of patients and plasma samples included in the total study population as well as sub analyses. Patients with MT data from previously published studies were excluded from the study population if no plasma samples were available for ST analysis or there was no target for ST analysis. Further, patients were excluded if not UICC stage II-III. Two patients were excluded as all ctDNA analysis failed ST quality control (QC). The study population included 112 patients and 373 paired plasma samples, all of which were included in concordance analysis. To assess preoperative sensitivity, only patients with analyzed samples collected before surgery were included. For postoperative analysis, patients were included if they had a plasma sample collected within 60 days after surgery and before start of adjuvant treatment (if applicable). For inclusion in the serial analysis, we required at least two samples to have been collected after EoT and before recurrence. With the exception, that we included recurrence patients with just one sample, if they recurred within 4.5 months after the operation. This was motivated by our 3 month sampling interval which prohibits collection of two samples prior to recurrence. An overview of serial samples is presented in Supplementary Figure S3. A subset of patients from the serial analysis was included in lead time analysis. These were recurrence patients with ctDNA detected by both ST and MT approaches. ACT=Adjuvant chemotherapy; EoT=End of Treatment (defined as after adjuvant treatment or after surgery, in case of no adjuvant treatment); MT=Multi-target, ST=Single-target. Analysis endpoint defined as end of radiological follow up or time of disease recurrence.
