# Supplementary material for: Comparing single‐target and multitarget approaches for postoperative circulating tumour DNA detection in stage II–III colorectal cancer patients
Source: Mol Oncol. 2022 Aug 18;16(20):3654–65. doi: 10.1002/1878-0261.13294 (PMC9580876; doi:10.1002/1878-0261.13294)
Supplement: Supplementary file 2 — Fig. S2. Example of positive and negative test result using single‐target ddPCR against the KRAS_c.35G>T_p.G12V mutation. [file MOL2-16-3654-s001.pdf]

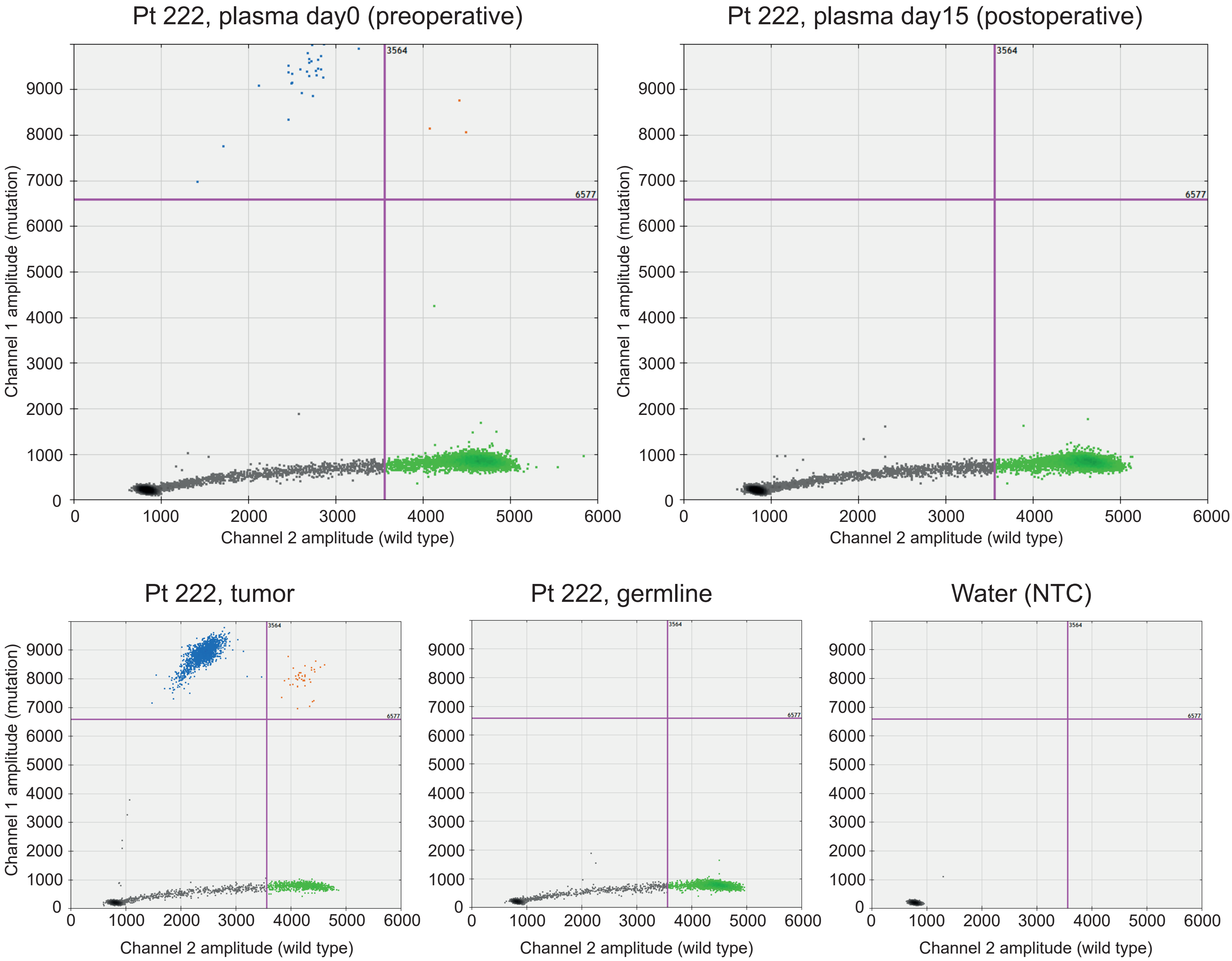

Supplementary Figure S2 – Example of positive and negative test result using single-target ddPCR against the KRAS_c.35G>T_p.G12V mutation. Example given for preoperative (testing ctDNA positive) and postoperative (testing ctDNA negative) plasma DNA samples. Results also shown for the patient's tumor DNA (positive control), the patients germline DNA (from buffy coat, negative control), and a water sample (no-template control). Screenshots from the QuantaSoft software (v1.7.4, Bio-Rad).
