# Supplementary material for: Comparing single‐target and multitarget approaches for postoperative circulating tumour DNA detection in stage II–III colorectal cancer patients
Source: Mol Oncol. 2022 Aug 18;16(20):3654–65. doi: 10.1002/1878-0261.13294 (PMC9580876; doi:10.1002/1878-0261.13294)
Supplement: Supplementary file 3 — Fig. S3. Overview of samples included in serial analysis and lead‐time analysis. [file MOL2-16-3654-s005.pdf]

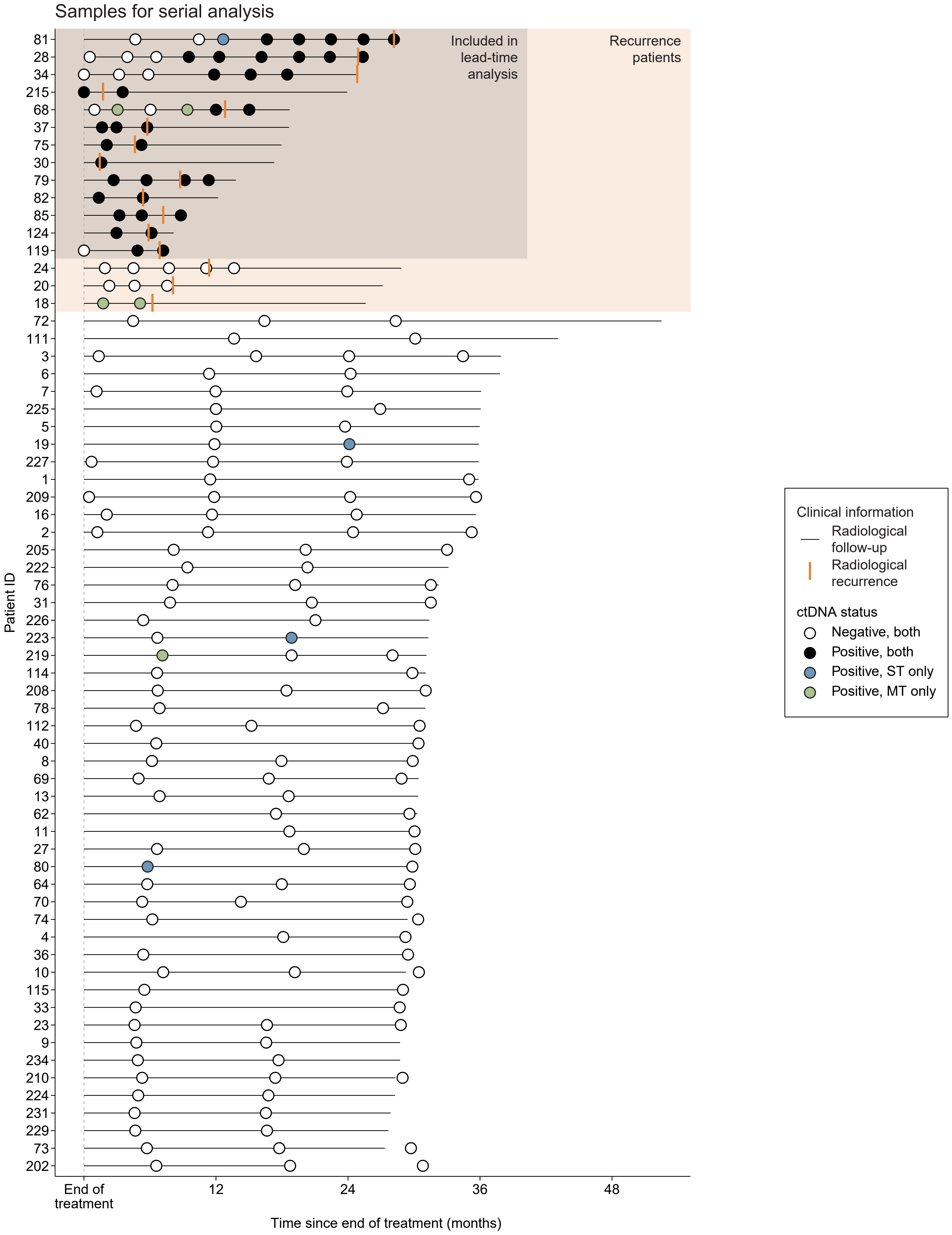

Supplementary Figure S3 – Overview of samples included in serial analysis and lead-time analysis. Samples were included in serial analysis if they were collected after end of treatment (EoT). EoT was defined as surgery if the patient did not receive adjuvant chemotherapy (ACT); otherwise, EoT was defined as end of ACT. In recurrence patients (orange shading), only samples collected until the time of recurrence (defined as within three months of recurrence) were included in the analysis. Only patients with at least two serial samples were included, unless recurrence was diagnosed shortly after EoT (within 4.5 months), where multiple sampling would not have been possible. Patients were included in lead-time analysis (grey shading) if ctDNA was detected in serial samples from recurrence patients by both ST and MT detection approaches.
